# Biocompatible and Biomaterials Application in Drug Delivery System in Oral Cavity

**DOI:** 10.1155/2021/9011226

**Published:** 2021-11-13

**Authors:** Lotfollah Kamali Hakim, Mohsen Yazdanian, Mostafa Alam, Kamyar Abbasi, Hamid Tebyaniyan, Elahe Tahmasebi, Danial Khayatan, Alexander Seifalian, Reza Ranjbar, Alireza Yazdanian

**Affiliations:** ^1^Research Center for Prevention of Oral and Dental Diseases, Baqiyatallah University of Medical Sciences, Tehran, Iran; ^2^School of Dentistry, Baqiyatallah University of Medical Sciences, Tehran, Iran; ^3^Oral and Maxillofacial Surgery, School of Dentistry, Tehran University of Medical Science, Tehran, Iran; ^4^Department of Oral and Maxillofacial Surgery, School of Dentistry, Shahid Beheshti University of Medical Sciences, Tehran, Iran; ^5^Department of Prosthodontics, School of Dentistry, Shahid Beheshti University of Medical Sciences, Tehran, Iran; ^6^Science and Research Branch, Islamic Azad University, Tehran, Iran; ^7^Faculty of Pharmacy, Tehran Medical Sciences, Islamic Azad University, Tehran, Iran; ^8^Nanotechnology and Regenerative Medicine Commercialization Centre (NanoRegMed Ltd.), The London Bioscience Innovation Centre, London, UK; ^9^Department of Veterinary, Science and Research Branch, Islamic Azad University, Tehran, Iran

## Abstract

Biomaterials applications have rapidly expanded into different fields of sciences. One of the important fields of using biomaterials is dentistry, which can facilitate implantation, surgery, and treatment of oral diseases such as peri-implantitis, periodontitis, and other dental problems. Drug delivery systems based on biocompatible materials play a vital role in the release of drugs into aim tissues of the oral cavity with minimum side effects. Therefore, scientists have studied various delivery systems to improve the efficacy and acceptability of therapeutic approaches in dental problems and oral diseases. Also, biomaterials could be utilized as carriers in biocompatible drug delivery systems. For instance, natural polymeric substances, such as gelatin, chitosan, calcium phosphate, alginate, and xanthan gum are used to prepare different forms of delivery systems. In addition, some alloys are conducted in drug complexes for the better in transportation. Delivery systems based on biomaterials are provided with different strategies, although individual biomaterial has advantages and disadvantages which have a significant influence on transportation of complex such as solubility in physiological environments or distribution in tissues. Biomaterials have antibacterial and anti-inflammatory effects and prolonged time contact and even enhance antibiotic activities in oral infections. Moreover, these biomaterials are commonly prepared in some forms such as particulate complex, fibers, microspheres, gels, hydrogels, and injectable systems. In this review, we examined the application of biocompatible materials in drug delivery systems of oral and dental diseases or problems.

## 1. Introduction

**Figure 1:**
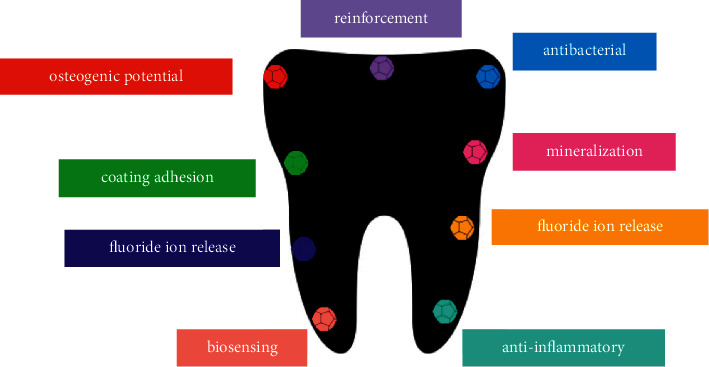
Some of the most important properties that can be enhanced in dentistry [12].

The first part of the digestive system is the mouth (oral cavity). It comprises various structures containing gum (gingiva), teeth, soft and hard palate, tongue, mucosal membrane of the cheek's inner surface, lips, and their supporting tissues. The oral cavity is a complicated niche for the colonization of more than 700 species of microorganisms that are responsible for oral health. The oral microbiota helps balance and stabilize oral microecology and prevent pathogenic microorganisms from growing [1]. Some factors, including systemic diseases, change of diet, and inadequate oral hygiene, change the oral microbiota composition that plays an important role in oral microecology dysbiosis and diseases related to different oral microbiota diseases such as oral infections. In addition to injuries, the most common dental problems and oral diseases involve oral infections, oral malignancies, dental caries, and periodontal diseases [2]. However, recent DNA and RNA studies have shown a wide range of ecosystems where *Streptococcus mutans* are considered a tiny fraction of the bacterial species. Thus, multiple actions of microorganisms that have collective or synergistic effects on expanding or initiating the cavity that exerts antimicrobial treatment are not adequate for caries and multiple microbial disorders, which do not obey classical Koch's postulates [3]. Also, oral infections such as peri-implantitis, periodontitis, dental caries, and oral candidiasis occur due to microbial dysbiosis rather than specific species of bacteria. Moreover, it has been clarified that systemic health is dependent on oral health [2]. Promoting tissue regeneration, inflammatory responses, and prevention of growing bacteria are closely associated with drug therapy. Two important ways of drug administration are local administration and systemic drug delivery, which might cause several other problems. For instance, in peri-implantitis and periodontal diseases, antimicrobials, such as nitroimidazole, doxycycline, and beta-lactam antibiotics, have been administered systemically [4]. Systemic antimicrobials might be important reasons for systemic adverse reactions, antimicrobial resistance, and dysbacteriosis. Furthermore, the delivery of the antimicrobials to the oral lesion is limited because of the systemic circulation. This points out the importance of drug delivery systems in oral and dental problems recently. Drug delivery systems are composed of transporters and correlated therapeutics that usually carry and release selected therapeutic compounds or bioactive agents to a precise area at specific rates in vivo [5]. Local delivery of therapeutic agents has many manifest advantages in comparison with systemic administration of drugs, targeting certain areas with minimum systemic adverse reactions [6]. Local delivery systems for dental and oral diseases have drawn great attention because these are common ways to use in humans. At least once, every individual suffers from dental caries or experiencing some gingival problems. Furthermore, oral malignancy is one of the most frequent kinds of cancer. Traditional approaches are costly and the fourth most expensive disorder to treat in most industrialized countries. Several oral disorders gradually occur, and therefore, these diseases require chronic therapeutic approaches. Moreover, approximately most oral disorders may be locally treated, without wide systemic distribution or ingestion. Nevertheless, delivery of intended concentration into the oral cavity has a few limitations, such as restriction of the route of administration value and taste sensation [7]. Oral infectious diseases include abundant microbial species that locate and colonize in certain areas, convening into biofilms, and accordingly become periodontal tissues or dental destructions. Thus, there is a necessity for novel systems that conduct individual antimicrobial products without cytotoxicity. Currently, new drug delivery systems have been examined in dentistry with mentioned characteristics based on biomaterials [8]. One of the strategies to support healthy tissue formation composes the mixture of synthetic or natural polymers with different materials to promote integration into dental tissues, increase interactions between cells, and contribute degradation kinetics and tunable material features in regenerative dentistry [9]. For example, materials of composite filling have been utilized to release fluoride ions and various types of biocompatible materials, containing hydrogels, nanofibers, nano- and microparticles, and porous scaffolds, have been used in several circumstances, such as teeth regeneration, salivary gland rehabilitation, oral infections, and other dental problems [10]. Devices and formulation attributed to biomaterials protect sensitive drugs and refine the retention time of active ingredients from days to months with due attention to the improvement of results of therapeutic approaches, individual compliance, and comfort. There have been developed to improve drug delivery system strategies through different polymers and micro- or nanoparticles to prohibit the formation of biofilm [11] (Figure 1). This review study discussed biomaterials effects and their drug delivery application in different dental problems.

## 2. Methods and Materials

The present review was conducted by adopting databases: PubMed, SciFinder, ScienceDirect, and Google Scholar. Extensive bibliographic research has been performed using, in the first part of the research, the following keywords “drug delivery system”, “biomaterials”, “dental diseases”, “oral cavity”, and “biocompatible” and each of related materials. 487 articles were found, of which 81 were approved for the writing phase and 135 articles were rejected because they were not relevant due to emphasis on the delivery system, they have used repeated information, or they did not have documented data. The selected material focuses on the evaluation of polymeric substances, administration, delivery system properties, and their effects in dentistry and medicinal fields. Articles and patents in the English language have been chosen. The second phase of bibliographic research was carried out focusing on each biomaterial and biocompatible compound. The following keywords are selected, “Chitosan”, “Calcium phosphate”, “Alginate”, “Hyaluronic acid”, “Gelatin”, “Silver nanoparticle”, “Titanium”, and “Gold nanoparticle”. The process of bibliographic research has been conducted from June 2021 to July 2020 comprehending works from 1999 to 2021.

### 2.1. Biocompatible Materials in Drug Delivery System

Biocompatible materials and systems demonstrated their potential in dental and oral diseases. Overall, biomaterial conducts a part of a system that contacts directly with biological tissues and improves or replaces any organ, tissue, or body function [13]. Also, biopolymers including hyaluronic acid, gelatin, chitosan, and collagen are studied in dentistry due to their capacities, such as native tissue. Many strategies have shown the different technological and biological properties to produce nano- or microcomposites. In particular, there is an attractive and emerging interest in biomaterials used as materials or delivery systems in dentistry [14]. Some recent research of biocompatible and biomaterials as carriers in drug delivery systems is mentioned in Table 1.

### 2.2. Natural Polymeric-Based Formulation for Drug Delivery in Dental Problems

Various methods have been established to target antibiotics from materials of implants to certain areas. Some of the investigated strategies are associated with biocompatible materials and are helpful for dentistry. Several studies emphasized using biocompatible alloys with other biomaterials such as silica sol-gel, polylactic acid, chitosan, and gelatin because these are absorbable drug carriers [32]. As degrades of coating, infections are eliminated, and surfaces of implants remain to attain osseointegration theoretically. Moreover, antimicrobial coatings have a promising effect against a broad range of bacterial species and eradicate infections with no resistance of any strains. Biomaterials can deliver antibiotics at the interface of implant-tissue, systemic antibiotics in conjunction and develop a novel treatment system for eradicating persistent biofilm infections [33]. Drug delivery systems are also used to improve controlling of drug release, individualized aim area, prolonged time of contact, reduction of dose, and times of administration. Also, drug delivery systems are suitable transporters to carry active ingredients, genes, peptides, proteins, and vaccines. Drug-loaded natural polymeric forms provided with various biodegradable biomaterials, such as alginate, chitosan, calcium phosphate, gelatin, hyaluronic acids, have promising usage as drug delivery systems [34].

### 2.3. Chitosan as Natural Polymeric Carrier in Oral Cavity Delivery System in Dental Diseases

**Figure 2:**
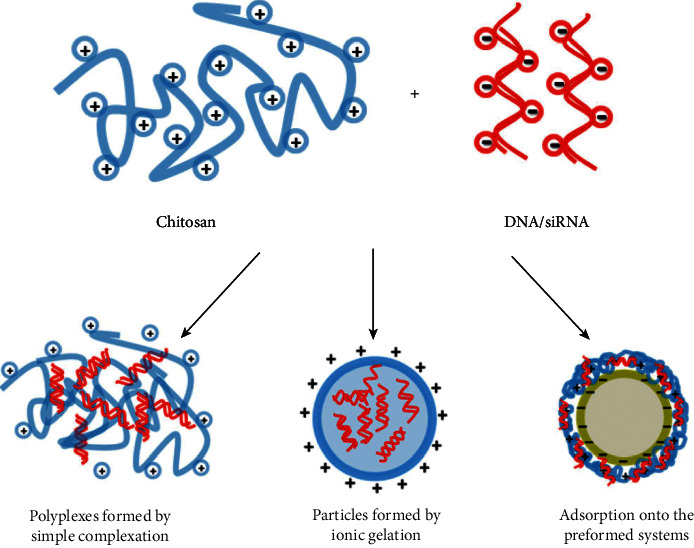
Chitosan-based particulate formulations following various strategies [40].

Chitosan has been known as a natural polymer conducted as a drug carrier due to its degradable capacity, nontoxicity, and high biocompatibility. Chitosan can be used in different types of formulation following the targeted function and suitable administration [35]. In the structure of chitosan, the protonated amino groups on D-glucosamine enable bonding to negatively charge the mucosal layer through penetrating deep layers of epithelium and electrostatic interaction. Also, chitosan has been used as a carrier in different routes of administration, such as ocular, nasal, pulmonary, and buccal routes because of the mucoadhesiveness feature of chitosan [15]. However, chitosan has insolubility problems in the physiological surrounding as a disadvantage that impact drug delivery and change through chemical alteration containing thiolation, carboxymethylation, quaternization, and acetylation. Carboxymethylated chitosan has a high solubility in a broad range of mucoadhesiveness and pH and accordingly enhances the penetration of carried drugs. Various forms of drug chitosan-based carrier such as films, fibers, microspheres, sponges, gels, hydrogels, and nano/microparticles contributed to delivering antibiotics, growth factors, chemotherapy drugs, vaccines, and anti-inflammatory agents to aim cells [36]. Chitosan-based drug delivery systems have been conducted in different conditions of dentistry fields, such as tooth caries, endodontics, treatment of peri-implantitis, and periodontitis and are usually used for local anesthesia [37]. Findings have demonstrated the anti-inflammatory effects of chitosan particles on HGFs (human gingival fibroblasts) through downregulation of chemokines and cytokines, such as CXCL-8, TNF-*α*, and IL-1*β*. However, the anti-inflammatory effects of chitosan can change the dependency of various formulations and excipients. In addition, studies have shown the increment of cells viability and HGFs metabolic activities via chitosan nano/microparticles, which describe the significant chitosan role in the reconstruction of injured tissue, particularly gingival tissue. Chitosan-based carriers are classified into injectable devices, gels, films, fibers, and micro/nanoparticles [38]. Microspheres of chitosan, provided through clinical and thermal cross-linking, solvent evaporation, spray drying, and techniques of emulsion, are spherical forms with a wide range of diameters (10–1000 *μ*m) and are fabricated to include drugs or other treatment agents with uniform distribution in the matrix which are natural and polymeric. Microencapsulated therapeutic agents prepared protective effects against saliva digestive enzymes and controlled and sustained release in the subgingival area [39]. In addition, DNA/siRNA can be attached to the surface of chitosan nanoparticles using adsorption, ionic gelation, and simple complexation (Figure 2) [40].

**Figure 3:**
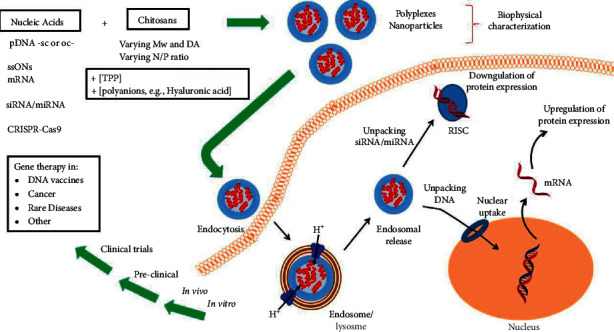
Chitosan induces a transgenic response and delivers nucleic acids intracellularly [40].

Moreover, surface and small size characteristics of chitosan particles affect their transportation by mucosal layers. Another limitation of chitosan-based drug nano/microparticles is the initial drug rupture that mitigates the encapsulation and stability efficacy which is solved by covering particles with other anionic biomaterials such as pectin, xanthan, alginate, gelatin, and hyaluronic acid [41]. One of the strategies to enhance the controlled release of drugs in acidic surroundings is polyelectrolyte complexation that additionally reduces toxicity in comparison with cross-linked chemical formulations. For example, the chitosan-alginate polyelectrolyte complexation delivery system provided through electrostatic interaction between alginate carboxyl groups and chitosan amino groups is more functional in particulate constructions containing micro/nanoparticles such as oral cavity administration of antibiotics [42]. Kumari et al. indicated that microspheres of sodium alginate/calcium/chitosan contemporaneous controlled release of doxycycline and ornidazole. Also, this finding showed antibacterial, biodegradability, and mucoadhesiveness of chitosan-alginate microspheres prepared by polyelectrolyte complexation [43]. Another procedure to prepare a microsphere/hydrogel carrier system is encircling the microspheres in hydrogels. This method has been elucidated to limit sudden releasing. Generally, various parameters impact the release of chitosan microspheres containing cross-linking density, drug content, chitosan concentration, and molecular weight [44]. And also, chitosan has been known as one of the attractive biopolymer in inducing a transgenic response (downregulation (siRNA or microRNA) or upregulation (pDNA, mRNA)) and delivering nucleic acids intracellularly (Figure 3) [40]. Meanwhile, some patients have the risk of allergic cross-reactions to chitosan or other dental varnishes because of seafood allergy or other allergic conditions to chitosan or other antimicrobial vehicles. Therefore alternative selection for transport drugs is carboxymethylcellulose, which can prohibit activation of *Streptococcus mutans*, simultaneously transport drugs to aimed receptors and present antimicrobial activity [45, 46].

### 2.4. Calcium Phosphate as Matrix of Antibiotics Delivery System in Oral Infections

Calcium phosphate materials (CP) are utilized differently in maxillofacial surgery and dentistry. Several methods of bone grafts and dental implantation have failed due to external infectious diseases and microbial biofilms. Antibiotics and CP mixture is responsible for decrement of infectious procedures percentage and improvement of situations. Delivery of antibiotics needs CP and doped vehicle, which has suitable mechanical and physicochemical characteristics as an apparatus of release [47]. CP is composed of a mixture of diverse calcium pyrophosphates (P_2_O_7_^−4^), metaphosphate (PO ^−3^), and orthophosphates (PO_4_^−3^). However, metaphosphate and pyrophosphates hydrolyzed in physiological environments become orthophosphates. Also, antibiotics are spread in the CP matrix for following controlled release as prevention or treatment is necessary [48]. Homogeneity of structure, grain properties (level of shape, agglomeration, and size), physicochemical features, and distribution of pores determine scattered antibiotics on the surface or within the matrix [49]. These properties show the release kinetics of antimicrobial agents from the procedure of the first phase of transfer that is controlled with surfaces while the second phase of release is sustained and correlated with matrix porosity and microstructure in a specific size and pores distribution and possibilities of functionalization [50]. Undertaking kinetics of release and related modification from the matrix to a physiological environment in drug delivery procedures requires explanation in vitro or anticipates interaction between antibiotic levels and time in biological fluids in vivo. Drug features are responsible for release procedures from CP and depend on their physicochemical and structural properties [51]. Stability of pH, chemical structure, the temperature of degradation, solubility, and molecular mass influence the desorption/absorption and linked procedures in the mechanism of release [52]. Vancomycin, gentamicin, doxycycline, ciprofloxacin, and amoxicillin are the most common antimicrobials that are used in bone infectious diseases. These antimicrobials spread out of the matrix with equivalent constant speed, but vancomycin migrates with lower speed due to the slower kinetics of release and higher weight molecularly [53]. Functional groups of antibiotics chemical structures such as -NH_2_, -OH, and -COOH are responsible for interaction with CP ions (Ca^+2^ and PO_4_^−3^) in the matrix. Also, these antibiotics are photosensitive in aqueous solution following the initial antibiotic concentration and pH including control difficulties in vitro [54]. Antibiotics may be involved in CP matrix in aqueous solution through different mechanisms such as dispersing in aqueous solution or solid phase or impregnation [55]. Antibiotics are absorbed via CP pores in the impregnation process. Insoluble antibiotics in aqueous environments are aligned with CP in solid state. In these conditions, antibiotics are scattered in the solid phase and then distributed in the matrix [47].

### 2.5. Alginate as Stabilizer Carrier in Oral Delivery Systems of Therapeutic Agents

Alginate (a linear polysaccharide) consists of *β*-D-mannuronic acid residues and alternative blocks of 1−4 linked *α*-L-guluronic. Cross-linking and gelation of alginate are attained through sodium ions exchange from guluronic acid with Ca^+2^ or other divalent cations. Some parameters such as bioadhesiveness, biocompatibility, mild gelation, and pH sensitivity have a significant role in designing a controlled delivery system made of alginate [56]. Alginate usually has been conducted as a carrier of peptides and proteins in oral delivery processes. The main challenge in proteins and peptides oral delivery is their degradation in acidic stomach conditions. The pH of the GI tract (gastrointestinal tract) ranges from acidic environments (stomach) to alkaline environments (colon and intestine), hence using alginate as a sensitive polymer to pH in oral delivery devices that carry peptides and proteins effectively [57]. Sodium alginate has been commonly used as an anionic polysaccharide due to its physicochemical, biocompatibility, and nontoxicity properties. Also, nanoparticle formulations containing alginate, which are known as bioactive agents, are suitable as a functional system in pharmaceutical properties [58]. Research has demonstrated that sodium alginate improved the selectivity of therapeutic agents release such as paclitaxel or doxorubicin in a certain area with enhancing safety. Moreover, sodium alginate, conducted as a stabilizer, and loaded silver nanoparticles indicated promising antibacterial activity in vitro [59].

### 2.6. Hyaluronic Acid as a Candidate Carrier for Antibacterial and Osteogenesis-Inductive Drug

Hyaluronic acid ( HyA ) consists of repeating disaccharides of *β*-glucuronic acid and N-acetyl-glucosamine. HyA hydrogel is diversely present in biological fluids. This is a major part of glycosaminoglycan structure that is correlated with other polysaccharides. HyA can bind to the surface of cells through cell receptors such as RHAMM and CD44. Findings have demonstrated that HyA can be used in the regeneration of bone and repairing cartilage drug delivery systems [60]. HyA-based scaffolds of bone regenerative are more active and compatible in biological environments with biomimetic mechanisms. HyA as a matrix constituent, particularly sulfated HyA, may promote modulation of cell behavior through many signaling pathways, which leads faster and better bone formations [61]. HyA-based carriers and scaffolds are formed into colloids or solid shapes. HyA can change the morphology of scaffold, improve the rate and efficacy of bone regeneration, and improve mineralization when it is a rigid material of scaffold that is accompanied by other components or therapeutic agents. In addition, HyA is versatile chemically by cross-linking and simple modification. Some physicochemical properties of HyA, such as viscidity, pH, rheological, and charge characteristics, can improve states for delivery or gelation [62]. HyA microspheres and hydrogels typically act as coating substances in osteogenesis that alter the surface of implants and improve osseointegration. HyA applications as a carrier have developed rapidly on a large scale in surgery implantation of dentistry and orthopedic conditions to recover organs impairment and related functions [60]. Several types of research indicated that hybrid HyA coatings can support the proliferation of osteoblast and following osteogenesis, antiadhesive properties, and cell affinity of unified materials such as collagen [62]. HyA is a substance with antibacterial effects. Although this property is not common, HyA still conducts bacteriostasis feature. Moreover, HyA is a hydrophilic structure because it can absorb aqueous solutions and is fairly appropriate for the adhesion of antibacterials [63]. The mixture of hydrogel carrier and some antibacterial or osteogenesis-inductive drugs, such as simvastatin and cortisols, known as osteogenic enhancer, is adequate in several models and will increase bone regeneration and healing effect for different clinical disorders such as oral infections after surgery [64]. Some methods play a significant role in the osseointegration of implant surgeries. HyA-based particulate and hydrogels formulation can attach to the surfaces of metallic implants and release bioactive constituents. Consequently, osseointegration and osteogenesis are improved. However, the precise mechanism of HyA on osteogenesis needs more investigation [62, 65].

### 2.7. Gelatin as Promising Carrier in Novel Drug Delivery Systems

One of the biocompatible materials, which is derived from collagen through alkaline or acidic hydrolysis mechanism, has rapidly been used in pharmaceutical industry and medical fields due to gelatin compatibility, biodegradability, and suitable ability in modification of amino acids. Gelatin-based hydrogels may be shaped by cross-linking in aqueous solutions. Consequently, the molecules accumulate, and conformational properties alter from random coil to triple helix [66]. Gelatin hydrogel has been used as a drug delivery system due to its properties in the stabilization, increasing absorption, controlled release, and transport of therapeutic agents to the aimed area. Also, gelatin electrical capacity is crucially associated with procedures of gelatin preparation, and the electrical properties could alter [67]. The gels are commonly cross-linked via carbodiimides or aldehydes to enhance their stability for other utilization in biomedical fields because of weak noncovalent bonds of this form and simply broken at 37°C temperature [68]. Gelatin has capacities to prepare poly-ionic mixtures with charged therapeutic components such as polysaccharides, nucleotides, proteins, and growth factors, which provide it as a great delivery carrier for a wide range of biomolecules [69]. Recent studies have demonstrated that gelatin-based hydrogel promotes the anabolic properties of W9-peptide on bone generation induced by BMP2 better than collagen I in a calvarial defect model [70]. Moreover, gelatin hydrogels incorporated with RhFGF-2 (recombinant human fibroblast growth factor-2) are implanted in the back subcutis of an animal (mice) model to improve the biological effects of RhFGF-2 and remain in hydrogel about 15 days after implantation [71]. Furthermore, it has been shown that the controlled release of bFGF (basic fibroblast growth factor) from gelatin-based hydrogels increases the biological actions to trigger bone regeneration. Kimura et al.'s findings proposed that gelatin as the carrier of the hydrogel can be effective in RhFGF-2 delivery. However, there are no significant differences compared to using collagen as a carrier of RhFGF-2 [25]. Also, Omata et al. found that gelatin-*β*-TCP (gelatin–*β*-tricalcium phosphate) composite is a candidate to use as a scaffold for bone regenerations that provides controlled release of bFGF [24].

### 2.8. Metallic-Based Formulation for Drug Delivery in Dental Problems

Nanoparticles have several properties that present better surface-to-volume ratio, spherical or shape rod, which is used in different fields of medical sciences, especially dentistry and dental surgeries. Nanoparticles are classified into various groups such as natural polymers, synthetic polymers, and alloys. Metallic nanoparticles such as titanium, gold, and silver, have been utilized as carriers in dental problems and other diseases because of their chemical, mechanical, physical, and optical characteristics [72].

### 2.9. Titanium

Titanium-based materials have been used generally in different fields of dentistry, although titanium-based materials have not sufficient bioactivity in orthopedic or dental bone-implant osseointegration. One of the prevalent substances used generally in tissue and bone engineering is TiO_2_ which has the capacity for the stimulation of adhesion of cells, migration of cells, wound healing, and osseointegration. The titanium-based implant increased superb stability and enhanced biocompatibility and became an important material due to its restorative actions in dentistry, especially contemporary dentistry [12]. TiO_2_ nanoparticles dramatically increase the certain surface of the nanotube (100 ± 10 nm diameter) and, therefore, improve the loading efficiency of anti-inflammatory drugs such as ibuprofen. Also, the efficiency of gentamicin-loaded TiO_2_ nanotubes release with chitosan-based matrix increased from 14 days to 22 days, separately [73]. Osseointegration interaction among bone and titanium enables the implant to resist masticatory load transmitted to the special structures and finally achieve acceptable permanent stability. Currently, zirconia, as one of the alternatives to titanium, has been proposed because of the same biocompatibility and mechanical advantages. Although implant-mediated drug delivery systems approve the benefits of the implant integration process, it does not have a longer life than conventional implants [74]. The implant-mediated drug delivery system is composed of a simple mechanism that is convenient and useful, and also, the drug is released and received into the targeted tissue without any pain. The parenteral route of administration, such as intramuscular, subcutaneous, and intravenous, have various superiority over the gastrointestinal manner, which is the most acceptable, economical, and suitable. These types of administration prepare high bioavailability, the rapid activity of drugs, and often continuous usage of intravenous injection [75]. In addition, TiO_2_ diamond-shaped nanoparticles are functionally loaded with doxorubicin and PEG chains. Doxorubicin is almost completely released in an acidic environment correlated with cancer cells. In vivo study on mice bearing H22 tumors demonstrated smaller volumes of tumors when nanocarriers were used in comparison with doxorubicin treatment without nanocarriers. The nanocarrier delivery system proposed fewer adverse reactions and used a lower concentration of drugs to enhance the accuracy of delivery mechanisms. Also, particulate formulation suggested a new mechanism in receiving anticancer agents and other drugs into certain areas in various tissues such as oral tissues [76].

### 2.10. Gold Nanoparticle as Promising Carrier of Drugs in Dental Problems

Gold-based nanoparticles have several applications in drug delivery systems of sensitive drugs, peptides, proteins, and genes. Gold nanoparticles have been taken highly by cells when the size of particles is between 20 and 50 nm, and toxicity appears for sizes ranging from 40 to 50 nm. These nanoparticles are used in the fields of periodontology, restorative dentistry, tissue and bone engineering, dental implants, and cancer diagnosis. Moreover, various shapes of gold nanoparticles have been synthesized by different mechanisms [77]. As abovementioned, gold nanoparticles play a major role in the rapid and early diagnosis of periodontal disease. There is a lack of periodontium supporting tissue, especially periodontal ligament, in periodontal disorder and endorse alveolar bone, which causes teeth mobility [72]. Essawy et al.'s findings suggested that gold nanoparticles increase cytotoxicity against cancer cells and, furthermore, perform as carriers of therapeutic agents. pH-resistant gold nanoparticles contain doxorubicin increased aggregation of gold nanoparticles in nuclei of cancer cells involving remarkable cellular apoptosis with no deleterious effects on the count of blood cells approved by in vivo and in vitro experiments [20]. Moreover, biodegradability and biocompatibility of colloidal gold nanoparticles and PLGA (polylactic-co-glycolic acid) loaded with methylene blue were examined against E. *faecalis* in infected root canals. Gold nanoparticles enhance anchoring and permeability and receive higher levels of photoactive substances. This remains to be elucidated whether compounds have greater antibacterial properties than conventional therapeutic approaches [78].

### 2.11. Silver Nanoparticle as Antibacterial Carrier of Antibacterial Drugs

One of the significant antibacterial elements is silver which is used commonly for its antibacterial activity. Antibacterial effects are according to dose-dependent cytotoxicity that has importance in managing the antibacterial agent concentration to eliminate bacteria on surfaces of implants with the least deleterious effects on targeting tissues. Hence, surface fabrication of antibacterial agents is prioritized in coating technologies due to its simple influence on implants surface composition [79]. For instance, silver nanoparticles-doped poly-ethylene-imine is composed of the efficient coating material of the antibacterial surface. Nevertheless, the exposure of bacteria to silver, including membrane, contributed progression of the silver-resistant microorganisms. The last studies demonstrated that the leaching of silver nanoparticles from storage products and packaging is still a significant health concern [80]. Metronidazole inhibits the synthesis of nuclei acid through DNA disruption [81]. Therefore, enhancing cellular uptake by using silver nanoparticles guaranteed the adequate concentration of the drug delivered to the targeted zone while this complex can reduce the resistance against antimicrobial agents. Also, metronidazole usually needs a long duration of administration to have an effect, and sometimes adverse reactions appear, such as metallic taste, loss of appetite, nausea, and vomiting [82]. Silver nanoparticles have inhibitory effects, which are correlated with bacterial cell disruption, inhibition of DNA synthesis, and permeability. Thus, the complexation of metronidazole and silver nanoparticles increases the action of metronidazole by enhancing the permeability of cells. Also, the organometallic complex has about two times more antimicrobial activity than its individual substances in both in vivo and in vitro studies [83].

### 2.12. Future Challenges

Drug delivery systems have been developed drastically, and their properties based on biomaterials have been expanded in different vital levels of treatment approaches in recent years. For instance, these systems enhanced the efficacy and decreased the adverse reactions in the treatment of oral infectious diseases or other dental problems. Scientists have examined various applications to inhibit bacterial growth, facilitate wound healing, prohibit biofilm formation, reinforce bone regeneration, and reduce inflammatory responses. Particularly, antifungal and antibacterial agents loaded biomaterials have been explored and demonstrated sustained bacteriostasis. Also, bone regeneration agents loaded in biomaterials have been studied in the acceleration of repairing hard tissues. Some other drug delivery systems are attributed to dental materials for modification processes. These findings indicate that biomaterials' effects in different forms show the importance of carriers in the prevention and treatment of oral diseases, such as infections, wounds, and even cancers. Some studies have elucidated that dual-functional delivery systems have synergistic influences. Therefore, drug delivery system efficacy is increased, and the necessary dosage of a drug is decreased. Also, there are several limitations in the field although research about biomaterials and their application in drug delivery systems greatly impacts oral medicine in various dental diseases. For more clinical application, in vivo and long-term research may be designed and studied. Also, multifunctional drug delivery following biomaterials, especially natural polymers, which can be combined in the therapeutic approaches of oral diseases, has a promising perspective. Additionally, the oral delivery system is a great pathway to receive the formulation to the optimized area. Although chitosan, hyaluronic acid, calcium phosphates, gelatin, alginate, silver, gold, and titanium are used in various conditions and forms for dental problems these days, long-term effects and reloading the systems in implants in oral diseases are still unsolved problems. The development of technology and biomaterials in the future would provide facilities for research on the improvement of drug delivery based on biomaterial systems optimized and used in clinical experiments.

## 3. Conclusion and Perspectives

With regard to increasing the knowledge about biomaterials and their utilization in dental problems such as peri-implantitis, bone regeneration, osseointegration, healing, dental plaque, and other related infections, different delivery systems have been schemed to improve the bioavailability and permeability and reduce the systemic side effects of the drugs or components such as antibiotics. These biomaterials include natural polymers, chitosan, alginate, calcium phosphate, gelatin, hyaluronic acids, and metal-based carriers such as titanium, gold, and silver. Changing the habits of using nonbiodegradable materials to biomaterials facilitates the control rate of drug release and receive the optimum biocompatible formulations, reduction of dosage frequency, and minimize the bacterial infections in the oral cavity. These systems and devices propose effective delivery systems for dental diseases therapy. Nevertheless, various forms of biomaterials carrier containing chips, microspheres, fibers, nanofibers, nanoparticles, and dendrimers should be examined more in clinical studies to find precise compounds to idealized the formulation and treatment used in dentistry.

## Figures and Tables

**Table 1:** Summary of biocompatible and biomaterials in different drug delivery systems.

Material	Compound	Dental problem	Method	Outcome	Ref
Chitosan/alginate polyelectrolyte complex film	Clindamycin phosphate	Periodontitis	Investigation of thickness, drug content, structure, release kinetics, and adhesion of formulations (in vitro)	Adhesiveness increased due to the increment of sodium alginate in contents and molecular weight of chitosan in complex films	[15]
Silver nanoparticles	Titanium	Peri-implantitis	Evaluation of antibacterial effects through disk diffusion tests (in vitro and in vivo)	Nanosilver improved healing process with its effect on surface properties	[16]
				Antibacterial activity of titanium enhanced by nanosilver particles	

Silver microparticles	Tetracycline/chlorohexidine	Regeneration of infected tissues	Evaluation of antibacterial activity of silver microparticles loading tetracycline/chlorhexidine on human dental pulp stem cells	Silver particles affect oral bacteria species by enhanced antibiotic delivery and membrane rupture through prohibition of protein synthesis	[17]
Gold nanoparticles	Doxorubicin	Gastric cancer cells	Evaluation of drug delivery of gold nanoparticles (in vitro)	Gold nanoparticles enhanced the efficacy of doxorubicin	[18]
Gold nanoparticles	*Salacia chinensis*	Implantation	Assessment of gold nanoparticles formation with UV-visible spectroscopy, X-RD, ICP-AES, AFM, Zetasizer, TEM and visual methods X-RD, ICP-AES, AFM, and TEM	Gold nanoparticles improved osteoinductive effect during dental implantation	[19]
Gold nanoparticles	Doxorubicin	Oral cancer	Evaluation of cytotoxicity in oral squamous cell carcinoma	Gold nanoparticles increased cytotoxic effect against oral cancer cells and induced apoptosis in cancer cells by increment of doxorubicin pH-resistant	[20]
			Investigation of resistance and pH-sensitivity of doxorubicin in hamster buccal pouch carcinoma model (in vivo and in vitro)		

Alginate–gelatin microspheres	Ciprofloxacin	*Pseudomonas aeruginosa* infection	Evaluation of matrix features by FTIR (Fourier transform infrared spectroscopy) and microsphere surface through SEM (scanning electron microscopy)	Alginate/gelatin matrix improve ciprofloxacin oral administration for infection diseases	[21]
Gelatin film	Ethanol extract of propolis	*Staphylococcus aureus* infection	Investigation of antimicrobial effects, adhesiveness, stability, and mechanical properties of the films in vitro	Films enhanced the stability and antimicrobial activities of the loaded extract in the oral mucosa	[22]
Gelatin films	Econazole nitrate	Mucosal candidiasis	Evaluation of the film features with X-RD	Easy to scale up and increase adhesiveness to mucosal tissue	[23]
Gelatin-*β*-tricalcium phosphate (gelatin-*β*-TCP)	Basic fibroblast growth factor (bFGF)	Bone regeneration	Evaluation of feasibility of gelatin-*β*-TCP	Gelatin-*β*-TCP controlled release of bFGF and improved bone regeneration	[24]
Gelatin hydrogel	Recombinant human fibroblast growth factor-2(RhFGF-2)	Hard tissue healing	Evaluation of feasibility of gelatin hydrogel to increase (RhFGF-2) induced osteogenic activities throughout distraction of rat mandibular (in vivo)	Enhanced hard tissue healing and treatment time following surgery	[25]
Alginate microparticles	Morin	Dental plaque	Investigation of physical properties of microparticles by SEM	Microbial viability and acidogenicity decreased	[26]
			Assessment of acidogenicity and microbial viability of composition		

Collagen	Tetracycline	Dental plaque	Investigate the resorbable collagen-based tetracycline via split-mouth design (in vivo)	Collagen improved antimicrobial agent delivery	[27]
Chitosan and hydroxypropyl methylcellulose (HPMC)	Cefuroxime axetil	Oral mucosal infections	Evaluation of release properties, surface morphology, adhesion, disintegration, water uptake, and mechanical strength of formulation (in vitro)	Chitosan increased antimicrobial activity, and HPMC increased control of drug release with appropriate adhesive properties, and mechanical strength	[28]
Gelatin and silk fibroin nanofibers	Doxycycline monohydrate	Oral mucosal infections	Investigation of physical properties of the gelatin and silk fibroin by using mouse fibroblast L929 cells (in vitro)	Addition of gelatin and silk fibroin increased surface wettability, nanofiber's diameter, bulk hydrophilicity, mass loss percentage, and reduced tensile strength, young's modulus, and porosity	[29]
Hydroxyapatite/titanium oxide	Antibiotics (amoxicillin, gentamicin tobramycin, cephalothin)	Oral postsurgery infections	Investigation of antibacterial effects of antibiotics was loaded into the hydroxyapatite by UV spectroscopy	Slow release of antibiotics loaded into the hydroxyapatite in implantation	[30]
Calcium phosphate	Vancomycin	Oral mucosal infections	Investigation of processing parameters on its degradation and vancomycin release	Improve control of drug release through managing the parameters	[31]

## Data Availability

There are no data as this is a review article.
